# T‐cell infiltration, contribution and regulation in the central nervous system post‐traumatic injury

**DOI:** 10.1111/cpr.13092

**Published:** 2021-06-29

**Authors:** Lvwan Xu, Xin Ye, Qingyi Wang, Bihan Xu, Jinjie Zhong, Ying‐ying Chen, Lin‐lin Wang

**Affiliations:** ^1^ Department of Basic Medicine Sciences, and Department of Orthopaedics of Sir Run Run Shaw Hospital Zhejiang University School of Medicine Hangzhou China; ^2^ Department of Neurosurgery Sir Run Run Shaw Hospital of Zhejiang University School of Medicine Hangzhou China; ^3^ Department of Basic Medicine Sciences, and Department of Obstetrics of the Second Affiliated Hospital Zhejiang University School of Medicine Hangzhou China

**Keywords:** central nervous system, clinical intervention, contribution, post‐traumatic injury, T cell

## Abstract

T cells participate in the repair process and immune response in the CNS post‐traumatic injury and play both a beneficial and harmful role. Together with nerve cells and other immune cells, they form a microenvironment in the CNS post‐traumatic injury. The repair of traumatic CNS injury is a long‐term process. T cells contribute to the repair of the injury site to influence the recovery. Recently, with the advance of new techniques, such as mass spectrometry‐based flow cytometry, modern live‐cell imaging, etc, research focusing on T cells is becoming one of the valuable directions for the future therapy of traumatic CNS injury. In this review, we summarized the infiltration, contribution and regulation of T cells in post‐traumatic injury, discussed the clinical significance and predicted the future research direction.

## Graphical abstract

The dynamic infiltration and the contribution of T cells in the CNS post‐traumatic injury. CD8^+^ T cells mediate neuronal apoptosis. Th1 cells and Th17 cells play pro‐inflammatory roles, while Th2 cells and Treg cells play anti‐inflammatory roles.

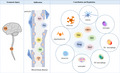

## 1 INTRODUCTION

Central nervous system (CNS) post‐traumatic injury usually results in irreversible impairment. It causes necrosis and apoptosis of neurons and glia due to the initial injury, followed by an expansion of secondary degeneration caused by the apoptosis of initially undamaged neurons.^1^, ^2^ There are two forms by which traumatic CNS injury may occur; specifically, traumatic brain injury (TBI) or spinal cord injury (SCI). TBI can lead to a significant increase in patient mortality and poor long‐term prognosis, leaving many survivors with long‐term disabilities.^3^, ^4^, ^5^, ^6^ SCI triggers a robust neuroinflammatory response and disrupts essential neuroimmune communication causing serious deficits in sensorimotor functions.^7^, ^8^, ^9^, ^10^ The incidence rate of SCI varies from regions to countries and increases when human activities are increasingly greater.^11^ In recent years, some research on the clinical treatment of CNS post‐traumatic injury has benefited patients, such as the improvement of surgical methods,^12^, ^13^ cell transplantation,^14^, ^15^ nerve segment transplantation^16^ and so on. The idea of the CNS having immune privilege is controversial,^17^, ^18^ but recent research has found that, within the meninges, there is a functional lymphatic system.^19^ In CNS post‐traumatic injury, when an immune response occurs, T‐cell‐mediated effects are also involved. T cells appear in different kinds and flavors.^20^ In this review, we divided them into CD4^+^ and CD8^+^ T cells according to their phenotype. CD4^+^ T cells are introduced into four subsets, including Th1, Th2, Treg and Th17 cells. Both CD4^+^ and CD8^+^ T cells are able to migrate into the CNS, causing inflammation and neurodegeneration and playing dual roles of damage and repair.^5^, ^21^, ^22^ The role of T cells in the CNS post‐traumatic injury is of great interest to us.

Recently, flow cytometry analysis showed that both CD4^+^ and CD8^+^ T cells could exist in the CNS. They were found in the naive brain compartment, including meninges, choroid plexus and parenchyma.^23^ More than 75% of CD4^+^ T cells were in the brain tissue rather than the meninges.^24^ However, the distribution of different types of T cells in different parts of the CNS after TBI or SCI is still unknown. The immune functions of T cells are both pathogenic and beneficial for the repair of the injured CNS.^25^ With advances in multicolour flow cytometric analysis,^5^ modern live‐cell imaging^26^ and other techniques, studies on animal models and humans have made new discoveries about T cells.^27^ Different subtypes of T cells have different functions, which may play a harmful or beneficial role in the repair of injury. To better understand them, we reviewed recent scientific research. The contribution of T cells on the process of repair and regeneration in the CNS post‐traumatic injury is the focus of this review. T cells might be targets to influence the recovery process.^28^, ^29^, ^30^ Clinical studies of T cells in the CNS post‐traumatic injury deserve further research in the future.

## 2 INFILTRATION OF T CELLS IN THE CNS POST‐TRAUMATIC INJURY

**FIGURE 1:**
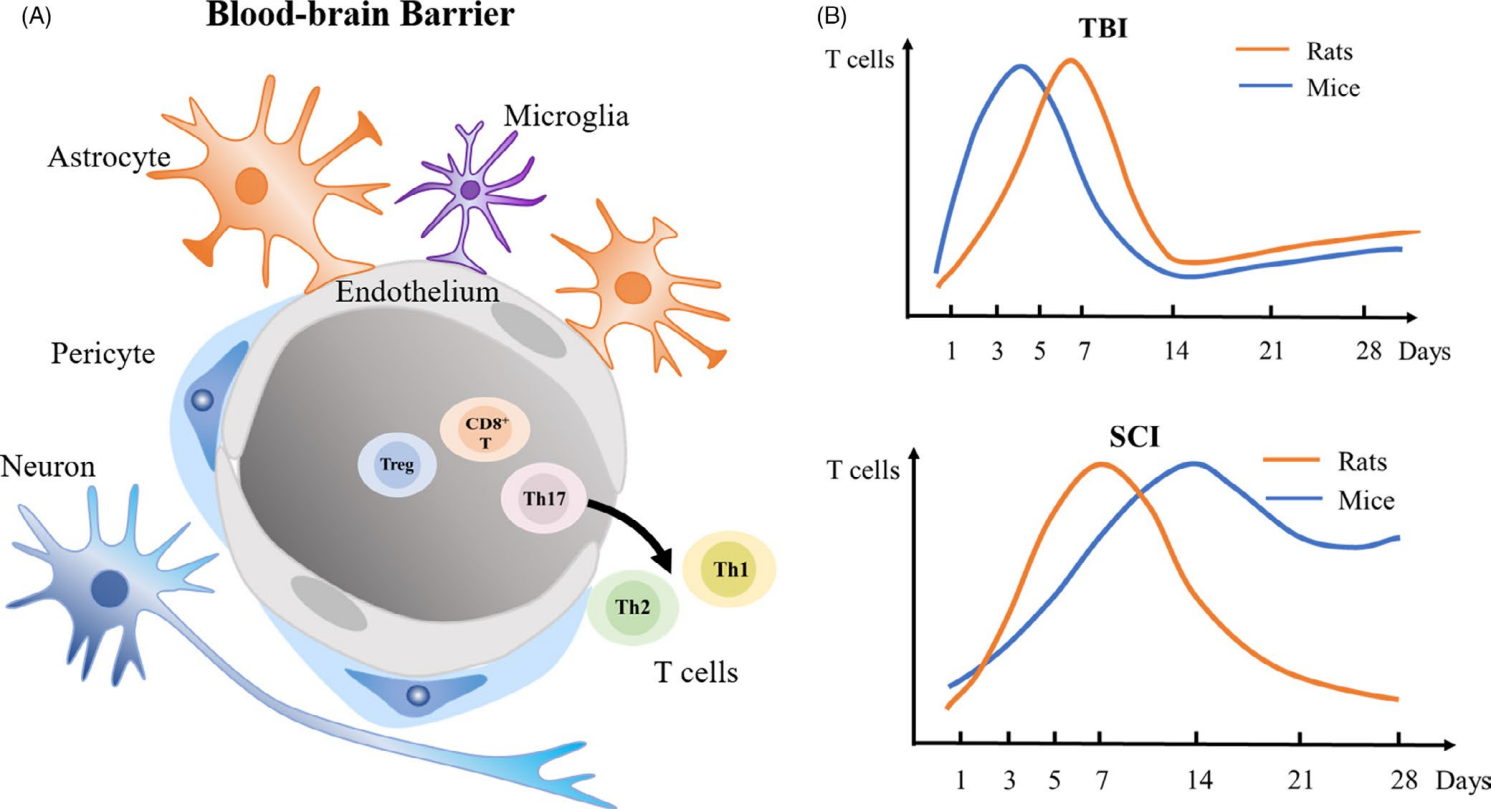
The dynamic infiltration of T cells in the CNS post‐traumatic injury. A, Schematic diagram of the blood‐brain barrier. After trauma, T cells cross the blood‐brain barrier and enter the CNS. B, The dynamic infiltration of T cells

How do T cells enter the central nervous system? It is reported that there is a functional lymphatic system in CNS.^19^ Recent studies have shown the existence of specialized meningeal lymphatic vessels. They allow the cells and soluble components of cerebrospinal fluid (CSF) access into the deep cervical lymph nodes instead of the nerve injury's sites,^31^, ^32^ which is likely to be a pathway for the antigen to enter the lymph nodes and activate T cells. However, whether lymph nodes other than the deep cervical lymph nodes participate in the formation of the lymphatic network of the CNS has not been reported. In addition, vertebral column lymphatic network has also been reported. It is connected to the lymph node and thoracic duct to drain the epidural space and dura mater. Peripheral sensory and sympathetic ganglia are also associated with it.^33^ The activation of T cells may also be related to neuroregulation and the brain‐gut axis, which needs to be confirmed by further studies. The communication network of the brain‐gut axis includes the parasympathetic and sympathetic branches of the autonomic nervous system, together with the enteric nervous system.^34^ In post‐traumatic stress, the damage to the brain‐gut axis results in a disturbance of the intestinal flora as well as a pro‐inflammatory immune response.^35^, ^36^, ^37^, ^38^ The transferred microbial antigens might encounter dendritic cells, presenting antigens to these lymphocytes and activating extra‐intestinal lymph nodes.^39^ Then, as the blood‐ brain barrier at the lesion site is destroyed, lymphocytes enter the parenchyma of the CNS (Figure 1A).^40^, ^41^

**FIGURE 2:**
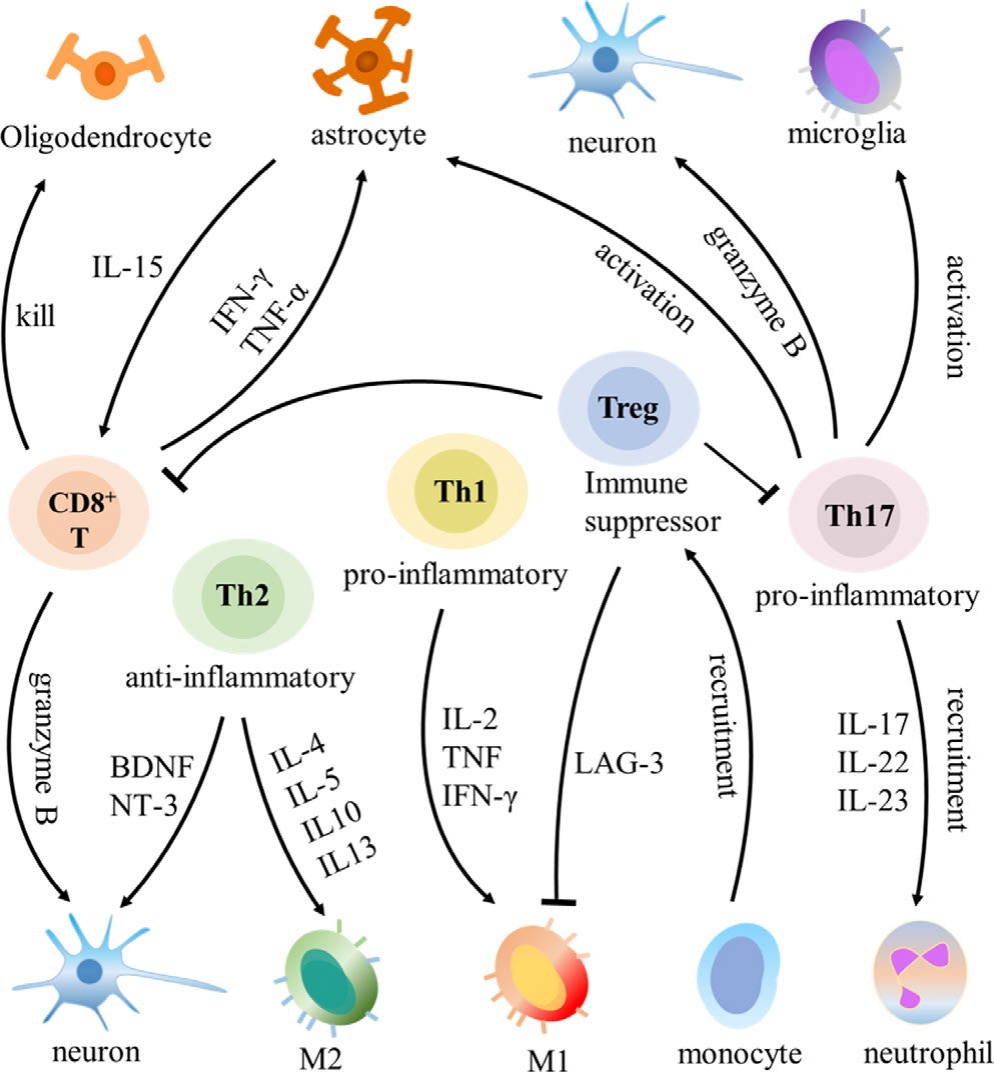
Different subtypes of T cells secrete different kinds of cytokines. CD8^+^ T cells mediate neuronal apoptosis. Th1 cells and Th17 cells play pro‐inflammatory roles, while Th2 cells and Treg cells play anti‐inflammatory roles. M1: classically activated macrophages; M2: alternatively activated macrophages

In term of the dynamic process of T‐cell infiltration, some studies have explored the timing of T‐cell infiltration to the injury site in the CNS post‐traumatic injury (Figure 1B). After TBI in rats, T‐cell number was significantly increased at 1 week. Then it was decreased. The proportion of CD4^+^ T cells decreased from the 1st week to the 4th week, and the proportion of CD8^+^ T cells was significantly reduced in the 1st week and then increased till the 4th week in a repetitive mild TBI rat model.^42^ After TBI in mice, T cells were found on days 3‐5 post‐trauma, whereas a reduced number of infiltrating T cells were detected on day 7 post‐trauma.^43^, ^44^, ^45^ After SCI, T‐cell number was highest on Days 3‐7 post‐trauma in rats but doubling on weeks 2‐6 in mice.^46^ Another study also showed that T cells produce an accumulation peak 1 week after SCI in rats.^47^ In the discussion that follows, we will elaborate on the contribution and regulation of different types of T cells in the CNS post‐traumatic injury based on findings in recent years (Figure 2, Table 1 and Table 2). We also identify future research directions at the same time.

**TABLE 1:** The effects of T cells on TBI

		Effects	Reference
CD8^+^ T cells		Mediate neuronal apoptosis	^55^
		Trigger chronic neurological and motor impairment and myelin pathology	^5^
		Cause long‐term neurological impairment	^28^
CD4^+^ T cells	Th1	Produce more IFN‐γ	^66^
		Cause white matter injury	^68^
	Treg	Inhibit overactive immune responses	^81^
		Balance the peripheral immune environment and improve functional outcomes	^83^

**TABLE 2:** The effects of T cells on SCI

		Effects	Reference
CD8^+^ T cells		Produce perforin to aggravate secondary spinal cord injury through destroying the blood‐spinal cord barrier	^2^
CD4+ T cells	Th1	Lead to neuron damage and demyelination	^63^
		Peripheral Th1 cells activate the epithelial blood‐cerebrospinal fluid barrier to produce molecules essential for the trafficking of leukocytes	^64^
	Th2	Support neuroplasticity	^40^
		Th2‐cell‐related cytokine IL‐10 promotes neuronal survival	^72^
	Treg	Their depletion can interfere with tissue remodelling in the subacute or chronic stages	^64^
	Th17	Aggravate neuroinflammation following SCI	^30^
		Inhibit locomotor function recovery	^92^

## 3 CD8^+^ T CELLS IN THE CNS POST‐TRAUMATIC INJURY

### 3.1 Characteristics of CD8^+^ T cells

Naive CD8^+^ T cells can proliferate and differentiate into a number of effector and memory cell types under stimulation. Effector CD8^+^ T‐cell‐associated transcription factors include PRDM1, TBX21 and EOMES, while memory CD8^+^ T‐cell‐associated transcription factors include LEF1, KLF2, FOXO1 and TCF7.^48^ Effector cells kill pathological cells and memory cells provide long‐term protective immunity.^49^, ^50^, ^51^ CD8^+^ T lymphocytes recognize antigens presented by MHC class I molecules through their T‐cell receptor (TCR).^52^ Activated CD8^+^ T cells secrete pro‐inflammatory cytokines, which include interleukin‐2 (IL‐2), tumour necrosis factor‐α (TNF‐α) and interferon‐γ (IFN‐γ).^53^ After TBI in mice, flow cytometric analysis shows a long‐term increase in effector/memory CD8^+^ T cells, which can express granzyme B and accumulate in the brain after TBI, inducing chronic neurological impairment and myelin pathology.^5^ After SCI in rats, CD8^+^ T cells were detected on the 1st day post‐injury, and then increased significantly and were markedly elevated on the 3rd, 7th and 14th day. Then the number of infiltrated CD8^+^ T cells was significantly reduced on the 21st and 28th day, and few could be found.^54^

### 3.2 Contribution of CD8^+^ T cells

The contribution of CD8^+^ T cells in the CNS post‐traumatic injury has been reported in several studies. After TBI, infiltrating CD8^+^ T cells in lesions were in close proximity to IL‐15‐expressing astrocytes, which subsequently release granzyme B; thus, activating caspase‐3 and cleaving poly‐ADP‐ribose polymerase and, ultimately, inducing neuronal apoptosis.^55^ CD8^+^ T cells also produce TNF‐α and IFN‐γ to stimulate astrocytes to secrete inflammatory cytokines.^56^ After SCI, CD8^+^ T cells can produce perforin. Perforin destroys tight‐junction proteins in the blood‐spinal cord barrier, allows infiltration of inflammatory cytokines and aggravates the injury.^2^

Most of the contribution of CD8^+^ T cells in the CNS post‐traumatic injury are negative. First, the accumulation of CD8^+^ T cells triggers chronic neurological and motor impairment and myelin pathology after TBI.^5^ Second, activated CD8^+^ T cells cause long‐term neurological impairment after TBI. Pharmacological exhaustion of CD8^+^ T cells can improve neurological outcomes and produce a neuroprotective Th2/Th17 immunological transition.^28^ Third, CD8^+^ T cells can aggravate the injury by producing perforin.^2^

### 3.3 Regulation of CD8^+^ T cells

How are the CD8^+^ T cells regulated at the wound site? On the one hand, the local cells, such as astrocytes, express IL‐15 and promote CD8^+^ T cells to release granzyme B.^55^ On the other hand, possibly because of their negative effects, the body has some way of inhibiting them to reduce their adverse effects on damaged tissue. Acute TBI and SCI activate the sympathetic nervous system, resulting in a large‐scale reorganization of the spinal sympathetic reflex circuit and immune dysfunction, then further upregulation of the expression of programmed cell death‐1, a classic sign of CD8^+^T cell failure, thereby impairing their function and leading to immunosuppression.^9^, ^57^ Additionally, changes in levels of hormones, such as cortisol, enhanced norepinephrine sensitivity after acute SCI, leading to the decreased activation of CD8^+^ T cells.^9^

So far, there are still many problems unsolved. For example, whether all the CD8^+^ T cells involved in CNS trauma are specific? Which factors activate or recruit them after trauma? And how they differ from other T cells in terms of gene expression? The exploration of these questions will enable us to gain further insights into the regulation of T cells. Considering the advantages together with the disadvantages of CD8^+^ T cells in the CNS post‐traumatic injury, we believe the future research direction is to regulate CD8^+^ T cells at different time periods post‐trauma through specific cytokines or targets, to obtain the maximum benefits with the lowest toxic effects.

## 4 CD4^+^ T CELLS IN THE CNS POST‐TRAUMATIC INJURY

### 4.1 Subsets of CD4^+^ T cells

When activated, CD4^+^ T cells will differentiate into distinct T‐helper (Th) cells. These subsets include Th1, Th2, Th17 and regulatory T (Treg) cells.^58^, ^59^ Although each subset functions differently, they are all involved in the CNS post‐traumatic injury. Here, each cell's unique function and regulation are summarized.

### 4.2 Th1 and Th2 subsets

#### 4.2.1 Characteristics of Th1 and Th2 cells

The characteristics of Th1 and Th2 cells have been extensively studied. Th1 and Th2 cells produce different cytokine groups and associate closely with different immune responses as two CD4^+^ T‐cell subsets.^60^ For the polarization of Th1 cells, they need IL‐12, T‐bet and the transcription factors STAT1 and STAT4.^61^ Cytokines related to Th1 cells are IL‐2, IFN‐γ and TNF‐α.^62^ They help produce classically activated macrophages (M1 phenotype) that have bactericidal activity and express a high level of inflammatory cytokines.^63^ For the polarization of Th2 cells, they need IL‐4, GATA‐3 and the transcription factor STAT6.^61^ Th2 cells are able to express neurotrophic factors, such as brain‐derived neurotrophic factor, as well as neuroprotective cytokines, such as IL‐4, IL‐5, IL‐10 and IL‐13.^40^ Th2 cytokines help produce alternatively activated macrophages (M2 phenotype), which exhibit enhanced phagocytic and anti‐inflammatory properties.^63^

#### 4.2.2 Contribution of Th1 and Th2 cells

Th cells respond to signals from the traumatic environment in the CNS, and they are necessary for neurological recovery following CNS traumatic injury.^21^ Th cells can function on neurons, influencing focal axonal injury and demyelination.^21^ Considering the contributions of Th cells, Th1 cells have been investigated extensively. By facilitating recruitment of resolving monocyte‐derived macrophages, Th1 cells are beneficial to the recovery following SCI.^64^ After axonal injury in the CNS, Th1 cells mediate neuroprotective autoimmunity.^65^

However, it is undeniable that the emergence and persistence of Th1 cells is accompanied by some problems. The expression of Th1‐associated cytokines, especially soluble cytokine IFN‐γ, increases in TBI and SCI.^66^ The role of IFN‐γ here remains controversial because genetic models have shown either beneficial or detrimental results.^67^ Although there are neurotrophic factors, the continuous expression of Th1 cytokines as well as the increased activation of M1 microglia or macrophages might lead to neuron damage and demyelination.^63^ For example, the activation of microglia and the infiltration of Th1 cells also cause white matter injury, characterized by diminished myelin basic protein and neurofilament NF200, reduced thickness of the corpus callosum and the external capsule and a decline in mature oligodendrocytes and oligodendrocyte precursor cells.^68^ Moreover, mice lacking Th1 cells have shown reduced macrophage infiltration to the traumatic site, which further supports that peripheral Th1 cells activate the epithelial blood‐CSF barrier to produce molecules essential for the trafficking of leukocytes at the initial traumatic stage.^64^

The role of Th2 cells is relatively positive. TBI demonstrated obvious changes from a Th1 to Th2 reaction.^69^ The transition from a Th1 to Th2 response is dominated by anti‐inflammatory factors, which creates a local microenvironment, induces polarization of microglia and macrophages and facilitate the protection of neurons.^70^ The increase in the number of Th2 cells is conducive to SCI repair, which may change the local microenvironment from mainly Th1 and M1 cells to one dominated by M2, Th2 and Treg cells.^71^ There is evidence that Th2 cells help support neuroplasticity.^40^ IL‐4 and IL‐10, as Th2‐cell‐related cytokines, can promote the survival and regeneration of nerve cells. They also improve the functional outcome of the CNS post‐traumatic injury.^72^ Th2 cells, in particular, are necessary for the induction of axonal sprouting by NT‐3, which is consistent with the view that they play neuroprotective and regenerative roles in the nervous system.^40^

#### 4.2.3 Regulation of Th1 and Th2 cell

The regulation of these two cell subsets can be divided into three parts: activation, infiltration and secretion of cytokines. First, polarization can be regulated by macrophages and myeloid TLR4. The shift to an immune response dominated by Th2 cells is caused by increasing the number of M2 macrophages.^70^ The polarization of Th cells after TBI mediated by activation of myeloid TLR4 established a link between a long‐term adaptive immune response and acute trauma in a mouse cortical impact model.^3^ Second, infiltration is associated with microglial secretion of chemokines such as CXCL10. Microglia secrete the pro‐inflammatory chemokine CXCL10 in response to the activation of STAT1 signalling. CXCL10 serves as the ligand and chemoattractant for CXCR3^+^ Th1 cells, stimulating T‐cell infiltration.^68^ Third, the secretion of cytokines is influenced by interleukins. For example, IL33 stimulates the production of Th2 cytokines. Then, the cytokines stimulate innate type 2 immune cells and may be beneficial to the recovery process in CNS injury.^73^, ^74^ In addition, because of Th17‐cell recruitment, IL‐7, which is mainly secreted by Th17 cells, was rapidly and massively increased in SCI.^30^ Anti‐IL‐7Rα monoclonal antibodies block the IL‐7 signalling pathway and are beneficial to the production of M2 macrophages. ^75^

Th1 and Th2 cells both play different roles in the CNS post‐traumatic injury, interact with each other and undergo dynamic changes at different stages of recovery. Together with other immune cells, especially macrophages, they regulate the microenvironment of injury tissue. In addition, questions such as their temporal and spatial changes at the traumatic CNS injury site, how they are activated or undergo apoptosis and their function pathway with neurons and astrocytes need to be further studied.

### 4.3 Treg cells in the CNS post‐traumatic injury

#### 4.3.1 Characteristics of Treg cells

CD4^+^ T cells are also able to differentiate into Treg cells, which can suppress the function of other T‐cell subsets. The development and function of Treg cells are dependent on the transcription factor Forkhead box P3 (Foxp3), and Treg cells usually produce high levels of CD25.^76^, ^77^ Therefore, they are thought to be a subset of CD4^+^ T cells that express Foxp3 and CD25.^78^ Under certain circumstances, Treg cells are able to differentiate into natural Treg (nTreg) cells and inducible Treg (iTreg) cells; nTreg cells are main cells to infiltrate into the CNS parenchyma post‐trauma.^21^ Treg cells not only secrete anti‐inflammatory cytokines such as IL‐10 and TGF‐β but also inhibit the activity of various immune cells.^79^ They also express the immune checkpoint receptor latent activation gene‐3(LAG‐3) to inhibit CX3CR1^+^ macrophages producing IL‐23 and IL‐1β.^80^ After TBI, the level of circulating Treg cells was increased compared to that in control group on the 1st day, decreased on the 4th day, increased on the 7th day, reached the peak on the 14th day and then decreased to the normal level on the 21st day in a clinical study.^78^

#### 4.3.2 Contribution of Treg cells

Treg cells work through acting on other T‐cell types. They suppress and, thus, manage effector T cells to inhibit overactive immune responses.^81^ Parenchymal Treg cells may react to the same chemokine cues as Th1 cells through upregulation of CXCR3, thereby contributing to the resolution of local Th1 response.^64^, ^82^ Besides, Treg cells are immune suppressors. If T cells fail to differentiate into Treg cells, the secretion of cytokines will be uncontrolled after TBI.^83^

Treg cells are thought to be potential targets for balancing the immune environment and improving functional outcomes in diffuse TBI. ^83^ It might be beneficial to control their reduction in the early stages after SCI. However, in the subacute or chronic stages, their depletion can cause problems by interfering with tissue remodeling.^64^

#### 4.3.3 Regulation of Treg cells

Treg cells, as a research hotspot for CNS repair after injury, maintain the stability of the body's internal environment after injury. Monocytes and various cytokines have been reported to modulate Treg cells. Infiltrating monocytes and macrophages were found to recruit Treg cells to the injured parenchyma.^64^ However, one study showed that purified monocytes from injured brains reduced the production of Treg cells in a mixed lymphocyte reaction in vitro.^3^ Neutralizing C‐C motif chemokine ligand 28(CCL28) or CCR10 decreases the recruitment of Treg cells and delays the healing of locomotor function in mice after SCI.^84^ Meanwhile, Sirtuin 4 inhibits AMP‐activated protein kinase signalling in Treg cells in vitro. It might be a mechanism that inhibits the anti‐neuroinflammatory activity of Treg cells in traumatic SCI in mice.^21^ Moreover, IL‐33 was reported to increase the expression of the spleen Treg‐cell marker Foxp3 24 hours after SCI in mice, indicating an expansion of Treg‐cell pools. However, subsequent flow cytometry did not show a higher number of Treg cells.^73^ Additionally, the depletion of Treg cells can increase T‐cell infiltration and astrocyte proliferation in acute experimental TBI.^85^

Although some studies have revealed the benefits of Treg cells in CNS post‐traumatic injury repair, it is still difficult to artificially regulate them properly.

### 4.4 Th17 cells in CNS post‐traumatic injury

#### 4.4.1 Characteristics of Th17 cells

In addition to the subsets mentioned above, Th17 cells have received much attention recently. They are characterized by production of inflammatory factor IL‐17.^86^ Th17 cells are defined with Th1‐like features due to their surface expression of the chemokine receptor CXCR3.^87^ Their differentiation requires IL‐6 and TGF‐β.^88^ They can secrete IL‐17, IL‐22 and IL‐23.^79^ Moreover, they participate in adaptive immunity and mediate the clearance of pathogens.^89^ The spatial and temporal characteristics of Th17‐cell infiltration post‐trauma in the CNS are unclear, which need to be explored in future studies.

#### 4.4.2 Contribution of Th17 cells

The role of Th17 cells remains controversial. At present, it is valuable to study the targeting of Th17 cells to various cells in the CNS post‐traumatic injury. There are some problems to be solved, such as whether chronically elevated production of Th17 cells functionally mediates delayed white matter loss after TBI. Thus, Th17 polarization may serve as a biomarker to prospectively identify patients at risk of chronic white matter injury after TBI. ^90^

#### 4.4.3 Regulation of Th17 cells

Th17 cells are restricted by Treg cells and the Th17/Treg balance is tightly controlled in vivo to restrict the detrimental effects of Th17 cells.^91^ Recent evidence shows the importance of the regulation of Th17 cells in the CNS post‐traumatic injury. Through regulating Th17‐cell recruitment and the level of IL‐17A, CCL20 aggravates neuroinflammation following SCI; thus, CCL20‐targeted therapy may become a hopeful clinical application in SCI.^30^ Additionally, after SCI, the knockout of Mir‐155 inhibits Th17‐cell differentiation and promotes locomotor function recovery.^92^ Using single‐cell RNA‐seq, Jellert T. Gaublomme et al proposed that the pathogenicity of Th17 cells might be manipulated by Gpr65, Plzp, Toso and Cd5l in an autoimmune encephalomyelitis model.^89^ This method can also be used to explore gene‐level regulation of Th17 cells in traumatic CNS injury.

## 5 CLINICAL SIGNIFICANCE OF T CELLS IN THE CNS POST‐TRAUMATIC INJURY

### 5.1 Correlation between T cells and clinical outcome

Recent evidence showed that T cells are closely associated with healing in patients with traumatic CNS injury. Reports indicate that T‐cell frequency in the blood of patients with brain trauma is lower than that of control group early after the insult. Sixty‐five percent of patients with severe TBI showed T lymphopenia on admission, and the reduction in T‐cell number was associated with a worse neurological outcome.^93^ Importantly, T‐cell levels gradually normalized during hospitalization.^94^ This finding suggests that there was a positive correlation between the circulating Treg‐cell level and the clinical outcome of TBI. Therefore, T‐cell levels may predict the progression for TBI patients and may be a target in the treatment of TBI.^78^ These results reflect the close association between T cells and the clinical prognosis of traumatic CNS injury. At present, it is worth exploring whether the change in T‐cell level over time is related to the CNS trauma degree or the prognosis of patients.

### 5.2 Intervention strategies targeting T cells

It is an important strategy to intervene or manipulate the process in the secondary injury. T‐cell regulation is one of the important targets, as many experiments in animal models have pointed to the potential clinical value of intervening with different types of T cells in the CNS after trauma. Based on previous reports, different subtypes of T cells are judged to be beneficial or detrimental to injury recovery. A crucial question is how to regulate T cells to draw on the advantages and avoid the disadvantages. The possible intervention strategies are drug therapy, cell transplantation and regulation of the gastrointestinal flora.

Pharmacological inhibition in CD8^+^ T cells can promote recovery in TBI mice, and offer a promising approach for patients with trauma to reduce long‐term disability.^28^ A competitive antagonist for MHC Class II–associated invariant peptide decreases peripheral splenic lymphocytes and reduces neuroinflammation and neurodegeneration after TBI in mice. ^29^ Although there is a lack of conclusive evidence to prove that the treatment has a significant effect, patients after SCI have already been given a large dose of glucocorticoids to reduce the immune response of the CNS.^25^ Pre‐treatment with pituitary adenylate cyclase‐activating polypeptide probably can protect against TBI by affecting peripheral T‐cell immune function.^95^ A study using mice model indicated that 3‐day consecutive Fingolimod application was a new immunomodulatory therapy. On the 3rd day after TBI, it could reduce the infiltration of T lymphocytes as well as NK cells, increase the percentage of Treg cells and the concentration of IL‐10. The use of Fingolimod regulates a variety of immune inflammatory reactions and improve neurological deficit, making it a possible way of treating secondary TBI.^96^ Anaesthesia drugs, such as propofol and dexmedetomidine, can induce an imbalance in Th1/Th2 cells during surgery of SCI patients and need to be used cautiously.^97^

Recently, there are some interesting reports showing the importance of the transplantation of T cells in immunomodulatory treatment in post‐traumatic CNS injury. First, a study showed that the transference of Th1‐conditioned cells but not Th2‐conditioned cells is beneficial to the functional recovery after SCI in IFN‐γ‐ and IL‐10‐dependent manner in mice. IL‐10 from Th1 cells is beneficial to functional recovery after SCI.^25^, ^98^ Although IL‐10 is mainly expressed by Th2 cells and inhibits the differentiation of Th1 cells, evidence suggests that some Th1 cells, such as IFN‐γ‐producing Tbet^+^ Th1, can also produce IL‐10 under certain conditions.^99^, ^100^, ^101^ Th1‐conditioned lymphocytes’ adoptive transfer can promote axon remodelling after SCI. In the injured spinal cord, there is little infiltration of adoptively transferred Th1 cells, indicating that Th1‐conditioned cells did not attack the CNS directly and, on the contrary, IL‐10 expressed by these cells promoted functional recovery.^98^ Second, the circulating Treg‐cell level may be a treatment target of traumatic CNS injury.^78^ Research has already found that Treg cells derived from human cord blood could regulate both the central and peripheral immune responses after TBI in rodent models.^102^ The interfering with the CCL28‐CCR10 axis may have a tremendous potential clinical significance in improving the recovery of SCI. CCL28 is upregulated after SCI and its upregulation relies on NF‐κB pathway. The spinal cord recruits CCR10‐expressing Treg cells through CCL28‐CCR10 axis, suppressing immune response and promoting recovery. ^103^

As for research on the regulation of the gastrointestinal flora for T cells, it was interesting to find that the number of Treg cells can be increased by VSL#3, a commercial medical‐grade probiotic that contains a large amount of lactic acid bacteria, after SCI.^104^ Daily prophylactic application of probiotics can reduce the Th1 / Th2 imbalance caused by severe TBI and reduce the hospital infection rate, which is mainly due to late infection.^105^ Although clinical trials for this aspect are lacking, as the technology advances, these are still important research directions and likely to enter the clinic in the future.

## 6 CONCLUSION AND FUTURE DIRECTIONS

Currently, the infiltration, function and regulation of T cells in the CNS post‐traumatic injury have been partially revealed. With the help of multicolour flow cytometric analysis and modern live‐cell imaging, the discovery of the lymphatic system of the CNS is very important to the study of T cells in the CNS post‐traumatic injury. It is suspected that there might be a group of CNS tissue‐specific T cells that enter the CNS through the lymphatic system at a certain time after trauma. Additionally, different types of T cells work together with glial cells and neurons to regulate the microenvironment of the damaged area and play a key role for a long period of time.

Currently, single‐cell sequencing studies have been conducted on T cells in CNS diseases, for example, Alzheimer's disease, and some results have already been obtained. Researchers found clonally amplified CD8^+^ T cells in the CSF of an Alzheimer's patient.^106^ In the future, their unique transcriptome could be discovered by single‐cell sequencing. Furthermore, to determine their fate more clearly and explore their spatiotemporal specificity, use of spatial transcriptomics is recommended.

In addition to the interventions mentioned above, we suspect that nutritional support,^107^ rehabilitation exercise, psychological intervention and other methods probably can regulate the T cells and the immune system, which may improve patients’ prognosis after CNS trauma injury. Considering the interaction between T cells and stem cells, in one aspect, we can use stem cells to regulate T cells. By regulating Th17/Treg‐relevant gene expression, inhibiting Th17‐related gene expression and promoting Treg‐related gene expression, peripheral blood mesenchymal stem cell transplantation‐related therapy will ultimately promote the functional recovery from SCI in rats.^108^

Therefore, how to maintain the proper balance of T cells is a substantial challenge for both the body itself and clinical treatment. After traumatic CNS injury, a delicate balance is supposed to be found by the intervention of the proportion and number of T cells in different subgroups, which can even mediate endogenous repair. This requires an in‐depth understanding of T cells. For example, how these T cells are activated after trauma? And how are the collaboration and competition among these T‐cell subsets and between them and other cells in the CNS? Repairing traumatic CNS injury can be a long‐term process. Therefore, T cells are not only related to the repair of the nerve injury site but also to the homeostasis of the body's whole immune system. Although T‐cell research has a bright future in the treatment of post‐traumatic injury in the CNS, there are still many questions to be answered if patients will be truly benefited.

## CONFLICT OF INTEREST

No competing financial interests exist.

## AUTHOR CONTRIBUTIONS

LWX and XY contributed equally to this work. LWX, XY, QYW and BHX wrote the draft manuscript. LWX and LLW designed the figures and tables. The manuscript was polished by LWX, XY, JJZ, YYC and LLW. All co‐authors (LWX, XY, QYW, BHX, JJZ, YYC and LLW) contributed substantially to revision and improvement of the manuscript. The authors read and approved the final manuscript.

## Data Availability

The data used in this study are available from the corresponding author upon request.
